# Dietary and circulating vitamin D and risk of renal cell carcinoma: a meta-analysis of observational studies

**DOI:** 10.1590/S1677-5538.IBJU.2020.0417

**Published:** 2020-08-25

**Authors:** Jing Wu, Nan Yang, Mingxin Yuan

**Affiliations:** 1 Second Affiliated Hospital of Harbin Medical University Department of Cadre Ward Harbin China Department of Cadre Ward, the Second Affiliated Hospital of Harbin Medical University, Harbin 150001, China

**Keywords:** Vitamin D, Carcinoma, Renal Cell, Meta-Analysis as Topic

## Abstract

**Objective:**

This meta-analysis is the first to evaluate the associations of circulating and dietary intake of vitamin D with risk of risk of renal cell carcinoma (RCC). Our findings showed that higher circulating vitamin D level and dietary vitamin D intake were associated with a reduced risk of RCC. The possible explanation might be attributed to the anti-inflammatory effect, inhibiting cell proliferation, inducing cell differentiation and apoptosis.

**Materials and Methods:**

We searched the MEDLINE, EMBASE, and Scopus databases from their inception points through December 2018 for observational studies. The pooled relative risks (RRs) with corresponding 95% CIs were calculated using random-effects or fixed-effects models. The Newcastle-Ottawa scale was employed to assess the quality of the included studies.

**Results:**

A total of 9 publications were included in this meta-analysis. An overall analysis of the highest versus lowest intake levels revealed that circulating vitamin D level was protectively associated with risk of RCC 0.76 (95% CI: 0.64-0.89, P=0.001), with no evidence of heterogeneity (I2=38.8%, P=0.162). In addition, dietary vitamin D intake was associated with a reduced risk of RCC (RR: 0.86; 95% CI: 75-0.99, P=0.030). Statistical heterogeneity was not identified (I2=28.8%, P=0.199). Subgroup analyses results showed the gender differences, and the associations were significant in results with women participants (RR: 0.70; 95% CI: 0.55-0.88) and case-control studies (RR: 0.80, 95% CI: 0.67-0.95).

**Conclusion:**

Higher circulating vitamin D level and higher dietary vitamin D intake both might be associated with a reduced risk of RCC. Further high-quality randomized controlled trials are required in the future to confirm our results.

## INTRODUCTION

Renal cell carcinoma (RCC) remains one of the most lethal urological malignancies, and approximately 80% of kidney malignant tumors are RCC (1, 2). Worldwide, RCC is the sixth most commonly diagnosed cancer in men and the 10th in women, accounting for 5% and 3% of all oncological diagnoses, respectively (3, 4). In addition, there are more than 270.000 new cases of RCC yearly, resulting in 116.000 deaths (5). Obesity, hypertension, family history, and smoking are well-established environmental risk factors of RCC (6-9). Moreover, research suggests that some dietary risk factors, including consumption of vegetables, fruits, oils, and meat products, play a role in RCC etiology (10-13). However, these environmental factors may not completely explain the etiology of this malignancy.

Vitamin D is produced in the body after exposure to UV radiation from sunlight and is also ingested from some foods and supplements (14). Furthermore, vitamin D is metabolized into circulating 25-hydroxyvitamin D [25(OH)D] in the liver, which is the primary circulating form of vitamin D (15). An increasing number of studies have explored concerning possible links between vitamin D and anticancer properties (16-18). Circulation vitamin D is primarily bound to vitamin D-binding protein (DBP), which can play a role in chemotaxis, macrophage activation, apoptosis and angiogenesis (19). Recent meta-analysis shows that higher DBP concentrations are associated with a lower overall risk of cancer (20). In addition, because the kidney is the major organ responsible for vitamin D metabolism and resorption, previous studies have examined whether vitamin D status may be related to kidney carcinogenesis (21-23). Data from a meta-analysis of 2 cohort studies and 7 case-control studies indicate that higher levels of circulating 25(OH)D can reduce kidney cancer risk by 21% (24). The vitamin D receptor (VDR) gene is reported to be associated with RCC risk (25).

Although some studies have indicated that the dietary intake and circulating level of vitamin D plays a beneficial role in preventing RCC, the limited evidence from individual studies is inconsistent. For example, a recent study that included participants taking prescriptions of vitamin D3 recorded a significantly increased risk of RCC in a Chinese population (26). Meanwhile, compared with the lowest quartile, no significant association between the highest dietary intake of vitamin D and RCC risk were observed among several studies (27, 28). In a large, nested case-control study, the findings also do not support the hypothesis that circulating vitamin D is inversely associated with the risk of kidney cancer (23). Thus, considering these inconsistent findings, we conducted a systematic review and meta-analysis to gain further insight into the association between RCC risk and dietary intake and circulating level of vitamin.

## MATERIALS AND METHODS

### 

#### Literature search

Two investigators independently searched MEDLINE, EMBASE, and Scopus databases from inception through February 2019. We used the following index terms: (“vitamin D” OR “25-hydroxyvitamin D”) AND (“renal carcinoma” OR “renal cancer” OR “kidney cancer” OR “renal tumors”). In addition, we searched reference lists of eligible studies and other potentially relevant review articles. No publication time and language restriction were applied.

#### Study selection

Studies were included in our analysis if they met the following criteria: (1) observational study, including prospective cohort, case-control, nested case-control, or case-cohort study; (2) studies that reported relative risk (RR), odds ratio (OR) or hazard ratio (HR) with corresponding 95% confident interval (CI) for dietary intake or circulating vitamin D and risk of RCC. We excluded the people that received prescriptions of vitamin D and used continuously. When more than one article reported the same study, only the complete publication was included. Two investigators independently assessed the titles and abstracts of the articles according to the inclusion criteria. In the full-text screening stage, the authors must reach a consensus to determine eligible studies. Any disagreements were resolved by discussion with a third author.

#### Data extraction and quality assessment

Data extraction was carried out by two authors independently. From each publication, we extracted the name of first author, publication year, country, study population, study design, number of cases, duration of follow-up, exposure details and adjusted ORs or RRs with 95% CI for the highest vs. the lowest level. Differences in data extraction between investigators were resolved by consensus.

We used the Newcastle-Ottawa scale (NOS) to assess the quality of the included studies, which addressed three main quality parameters: four items for selection, two items for comparability, and three items for exposure (case-control study) or outcome (cohort study) assessment (29). The score of each study ranged from 0-9 stars, and the study with a score of ≥7 was considered to have high quality.

### Statistical Analysis

As the prevalence of RCC was relatively low, ORs and HRs were considered as RRs (30). The pooled RRs with its corresponding 95% CI was calculated to assess the association of dietary intake and circulating vitamin D level with the risk of RCC. The potential heterogeneity between the included studies was assessed using the Q tests and I2 statistics. In the analysis, P <0.10 or I2>50% was considered to substantial heterogeneity, and the random-effects model was adopted. Otherwise, the fixed-effects model would be used. Subgroup analyses and meta-regression analyses were conducted to explore possible sources of heterogeneity and the impact of different variables on study results. If more than 10 studies were included, the sensitivity analysis was conducted to evaluate the stability of outcomes by using method of single study remove (31). Publication bias was assessed using visual inspection of funnel plots and Egger linear regression test. All statistical analyses were performed using STATA version 12.0 (StataCorp LP, College Station, TX, USA).

## RESULTS

### 

#### Literature Search

Based on the study selection criteria, we identified 873 and 1862 potentially articles in Medline and Embase, respectively. Furthermore, we identified 491 articles in reference lists of eligible studies. After removing the duplicates studies, 1981 potentially eligible articles were found. After title and abstract screening, 39 full text articles were assessed for eligibility. According to the inclusion and exclusion criteria, 22 articles were further excluded, because they were not observational studies. In addition, two articles did not report the risk of RCC, two were duplicated reports and two involved risk of other cancers. One study limited the cases to those who received prescriptions of vitamin D3 and used them continuously for at least 28 days, and did not reported the dietary vitamin D intake (26).

**Figure 1:**
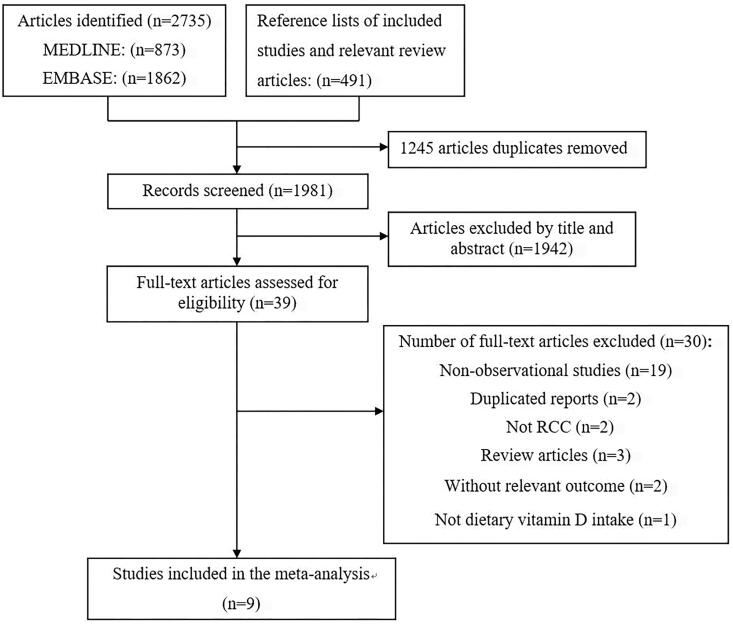
Flow diagram of literature searching and selection.

We excluded one case-control study which reported the same study in Finland (32, 33). Finally, nine articles, including four prospective cohort studies, two nested-case control and four case-control studies were included in this systematic review and meta-analysis. The flow diagram of study selection process is shown in Figure-1.

#### Study characteristics

Table-1 depicted the characteristics of 9 publications. All studies were published between 1996-2019 (23, 27, 28, 34-39). Three studies were conducted in the United States, one was conducted in seven centers in four countries in Central and Eastern Europe, and one each was conducted in Italy, France, Finland, China, and Denmark. A total of 2030 RCC cases and 3044 controls were enrolled in three case-control studies (27, 28, 37, 39). Joh et al. investigated the association between dietary vitamin D intake and RCC risk in the Nurse's Health Study (NHS) and the Health Professionals Follow-up Study (HPFS) cohorts of men and women in the United States, with follow-ups of 22 years, respectively (35). Seven studies involved both men and women, and four reported sex-specific results. The food consumption and dietary vitamin D intake from all the included studies were assessed using a food frequency questionnaire (FFQ). Table-1 lists the quality of the studies assessed using the NOS. Seven studies were scored 8 stars, one study was scored 7 stars, and one study was scored 5 stars in the quality assessment.

**Table 1:** Characteristics of included studies in meta-analysis.

First author, Year, Country	Study population	Study design	Sex, age, duration of follow-up	No. of cases/controls or cohort sizes	Exposure details	Highest vs. lowest analysis	RRs or ORs (95% CI)	NOS quality scores
Joh et al., 2013 (35), United States	NHS	Cohort study	Women, 30 to 55 years, 22 years of follow-up	72051	Dietary	Median 627 vs. 127 IU/d	0.81 (0.54, 1.22)	8
					Circulating	80nmol/L vs. 56nmol/L	0.60 (0.35, 1.02)	
	HPFS	Cohort study	Men, 40 to 75 years, 22 years of follow-up	46380	Dietary	Median 627 vs. 127 IU/d	0.8 (0.5, 1.27)	
					Circulating	70nmol/L vs. 50nmol/L	0.66 (0.40 to 1.09)	
Gallicchio et al., 2014, (23), in United States	VDPP	Nested case control	Men and women, 54 to 66 years, 2.2 to 10.9 years of follow-up	775/775	Circulating	≥100nmol/L vs. <25nmol/L	0.96 (0.43, 2.14)	8
Muller et al., 2014 (36), in France	EPIC	Nested case control	Men and women, 49 to 75 years, 6.7 years of follow-up	555/1092	Circulating	NA	0.82 (0.68, 0.99)	8
Karami, et al., 2008 (28), in Central and Eastern Europe	Hospital based study	Case-control	Men and women, 20 to 88 years	777/1035	Dietary	High (>66%) vs. Low (<33%)	0.81 (0.63, 1.3)	5
Wilson et al., 2009 (34), in Finland	ATBC	Cohort study	Men, 50 to 69 years, 3 years of follow-up	29133	Dietary	>6.8 vs. <3.5μg/day	1.5 (1, 2.3)	8
Bosetti et al., 2006 (27), in Italy	4 Italian areas	Case-control	Men and women, 24-79 years	767/1534	Dietary	Highest one vs. lowest one	Men: 0.92 (0.7, 1.21) Women: 0.6 (0.42, 0.88) Overall: 0.76 (0.57, 1.01)	7
Prineas et al., 1997 (38), in United States	LWHS	Cohort study	Women, 55-69 years, 8 years of follow-up	35192	Dietary	>476 vs. <3.5mg/day	0.81 (0.45, 1.45)	8
Mellemgaard et al., 1996 (37), in Denmark	Born and living in Denmark	Case-control	Men and women, 20 to 79 years	351/340	Dietary	Men: >3.3 vs. <l.6fg Women: >2.6 vs. <1.3mg	Men: 1.2 (0.6, 2.4) Women: 0.6 (0.3, 1.3)	8
Li et al.,, 2019 (39), in China	Hospital based study	Case-control	Men and women	135/135	Circulating	≥30 vs. <20	0.30 (0.13, 0.72)	8

**NHS** = Nurse's Health Study; **HPFS** = Health Professionals Follow-Up Study; **VDPP** = Vitamin D Pooling Project of Rarer Cancers; **EPIC** = EuropeanProspective Investigation into Cancer and Nutrition; **ATBC** = Alpha-Tocopherol Beta-Carotene Cancer Prevention Study; **LWHS** = Lowa Women's Health Study.

#### Circulating vitamin D level and risk of RCC

**Figure 2:**
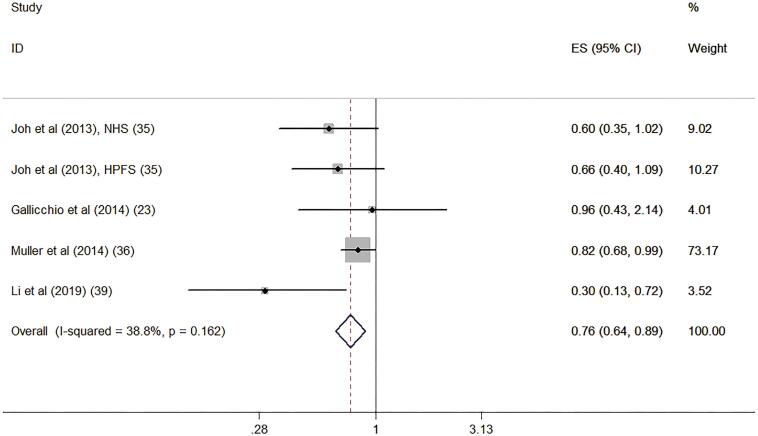
Forest plot for circulating vitamin D level and risk of renal cell carcinoma.

Four studies reported the association of circulating vitamin D level with risk of RCC (23, 35, 36, 39). Compared with the lowest quartile of circulating vitamin D level, the highest quartile of circulating vitamin D was associated with decreased risk of RCC (RR: 0.76, 95% CI: 0.64, 0.89, P=0.001). The statistical heterogeneity was not identified (I2=38.8%, P=0.162) (Figure-2).

#### Dietary vitamin D intake and risk of RCC

**Figure 3:**
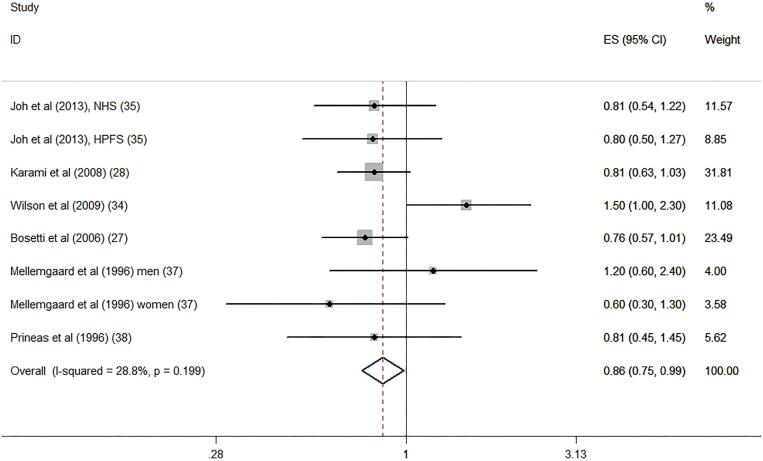
Forest plot for dietary vitamin D intake and risk of renal cell carcinoma.

Six studies reported the association of dietary intake of vitamin D level with risk of RCC (27,0 28, 34, 35, 37, 38). Figure-3 illustrates the forest plots of the pooled results for the association between the dietary vitamin D intake and risk of RCC. The pooled RR was 0.86 (95% CI: 0.75-.99, P=0.030). The statistical heterogeneity was not identified (I2=28.8%, P=0.199).

**Figure 4:**
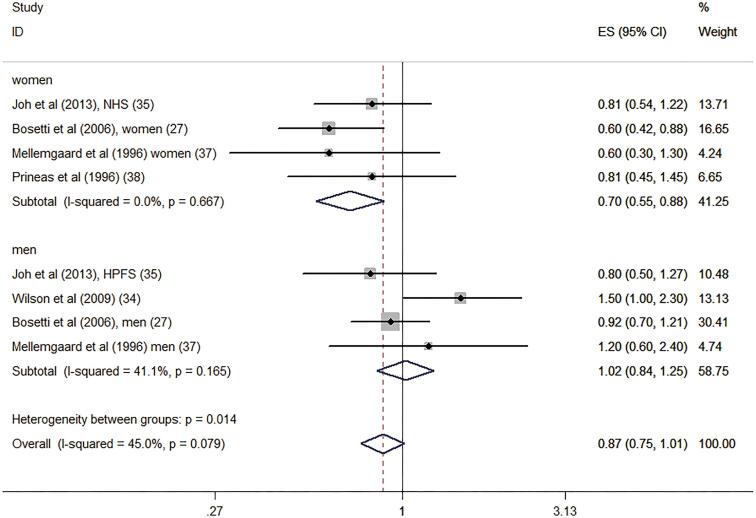
Subgroup analyses of dietary vitamin D intake and risk of renal cell carcinoma in different gender.

**Figure 5:**
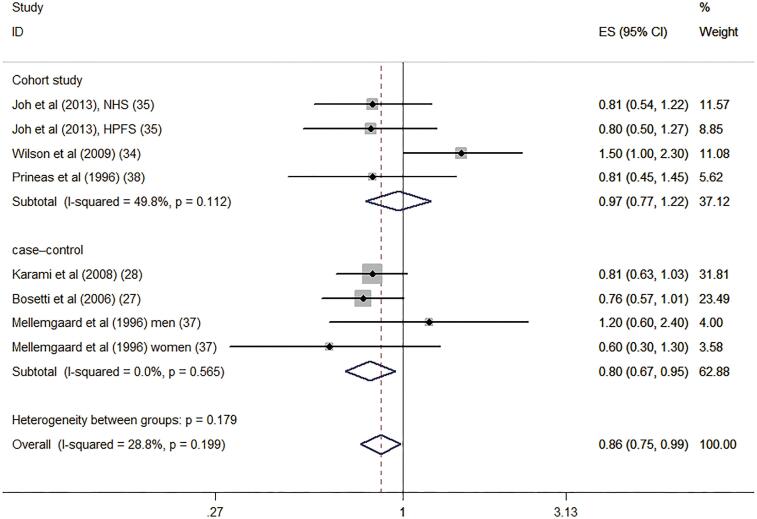
Subgroup analyses of dietary vitamin D intake and risk of renal cell carcinoma in different study design.

Subgroup analyses were performed according to the study design, location, gender participation, and quality as well as adjustment for alcohol and energy intakes (Table-2). Interestingly, the results indicated that the associations were significant among case-control studies (RR: 0.80, 95% CI: 0.67-0.95) and in results involving women (RR: 0.70, 95% CI: 0.55-0.88). However, the associations were no significant among cohort studies (RR: 0.97, 95% CI: 0.77-1.22) and men (RR: 1.02, 95% CI: 0.84-1.25). Confounders adjusted for alcohol and energy intakes also significantly altered the summary risk estimates. Figures 4 and 5 shows the subgroup analyses of dietary vitamin D intake and risk of renal cell carcinoma in different gender and study design.

**Table 2:** Subgroup analysis for dietary vitamin D intake and the risk of RCC.

Sub-groups		Num. of sub-groups	RRs (95% CI)	I^2^ (%)
Overall		7	0.85 (0.75, 0.97)	27.6
**Study design**				
	Cohort study	4	0.97 (0.77, 1.22)	49.8
	Case-control	3	0.80 (0.67, 0.95)	0
**Locations**				
	United States	3	0.81 (0.61, 1.06)	0
	Europe	5	0.88 (0.75, 1.03)	58.2
**Gender**				
	Women	4	0.70 (0.55, 0.88)	0
	Men	4	1.02 (0.84, 1.25)	41.1
**Study quality**				
	High (NOS score 6)	6	0.88 (0.74, 1.04)	36.3
	Low (NOS score ≤6)	1	0.81 (0.63, 1.04)	–
**Adjustment**				
	**Alcohol drinking**
	Yes	2	0.95 (0.75, 1.20)	85.6
	No	6	0.81 (0.69, 0.97)	0
	**Energy intake**
	Yes	6	0.97 (0.78, 1.22)	47.6
	No	2	0.79 (0.66, 0.94)	0

#### Publication bias

Visual inspection of funnel plots and Egger test showed no evidence of publication

bias for dietary vitamin D intake (P=0.583) and circulating vitamin D level (P=0.734).

## DISCUSSION

This meta-analysis is the first to evaluate the associations of circulating and dietary intake of vitamin D with risk of RCC. Circulating vitamin D level was protectively associated with risk of RCC. The results also indicate that a higher intake of dietary vitamin D might be associated with a reduced risk of RCC. Interestingly, subgroup analyses results revealed significant inverse associations between dietary vitamin D intake and RCC risk in the case-control studies but not in the cohort studies. In addition, we found that dietary vitamin D intake significantly reduced RCC risk among women participants.

The possible relation between vitamin D and RCC risk was first explored with regard to circulating 25(OH)D levels, which are accepted as a biomarker of vitamin D status. Previous studies reported that circulating 25(OH)D level was inversely associated with RCC risk (23, 40). Besides, similar to the findings of our meta-analysis, many researchers provided direct or indirect evidence for association between vitamin D and risk of RCC. For example, black people have a higher risk of RCC than white people in the United States, with vitamin D deficiency being common among black individuals (35). One cross-sectional study assessed the association between latitude and incidence rates of kidney cancer in 175 countries, finding that lower levels of UV irradiation were independently associated with higher kidney cancer risk (41). An occupational cohort study reported that high level of occupational sunlight exposure had a reduced kidney cancer risk among Swedish men construction workers (42).

Vitamin D is primarily metabolized within the kidneys, but the mechanism of vitamin D on RCC remains unclear. The possible explanation might be attributed to several reasons. First, the anti-cancer effects of vitamin D may derive from modulating cell proliferation and differentiation in immunity (17). In vitro and in vivo studies have shown that vitamin D and its metabolites, including 25(OH)D and 1, 25-(OH)D, could regulate the expression of many proteins and suppress tumorigenesis by inhibiting cell proliferation and inducing cell differentiation and apoptosis (28, 43). Second, vitamin D also has anti-inflammatory and anti-oxidative effects (44, 45), while inflammatory molecules can promote RCC progression (46). A recent in vitro experiment showed that vitamin D3 can significantly suppress NF-κB activation and adhesion molecules in RCC cells, which provide a mechanistic explanation for the association among low vitamin D status, local inflammation and increased expression of adhesion molecules among RCC patients (47). Third, serum 25(OH)D can bind to circulating DBP, which may directly play an anti-cancer role by scavenging extracellular actin and preventing actin release into the circulation to cause tissue injury or cell death. DBP may also activate macrophages and induces apoptosis (33). Many case-control and prospective studies have demonstrated that serum 25(OH)D is inversely associated with the risk of developing hypertension, diabetes and possibly obesity, which are also risk factors for kidney cancer (48, 49). Fourth, the anti-cancer effect of 25(OH) d may be related to the inhibition of tumor angiogenesis, invasion and metastasis in tumor (39). In addition, previous evidence showed that low serum 25(OH)D status was associated with IL-6/STAT3 hyper-activation among clear cell RCC (ccRCC) patients, and promote cell proliferation (50).

Our meta-analysis also found that dietary intake of vitamin D was associated with reduced risk of RCC. However, a prospective cohort study have yielded inconsistent results in Finland, which found no significant evidence of an interaction between dietary vitamin D intake or use of vitamin D supplements and RCC risk (34). By contrast, the results of risk estimates indicated an increased RCC risk associated with vitamin D intake. A previous study found that vitamin D supplementation might increase the risk of kidney stones among postmenopausal women (51). In addition, two studies reported by Mellemgaard et al. and Prineas et al. found that vitamin D intake did not reduce the risk of RCC (37, 38). These different findings might be explained by several reasons. First, studies have shown that low vitamin D status is mainly attributed to a lack of exposure to sunlight, with dietary vitamin D intake only accounting for a proportion of circulating 25(OH)D levels. Furthermore, dietary vitamin D intake may have been low in the populations of the included studies, meaning they were unable to achieve optimal concentrations. Research has shown that a 10ng/mL increment in serum 25(OH)D requires a supplement of 1500 IU/day of vitamin D (52). Interestingly, through conducting subgroup analysis in this study, we found that the associations between dietary vitamin D intake and risk of RCC were stronger for women and case-control study. However, the associations were no significant in men and cohort studies group. We conducted sensitivity analysis using single study remove approach, and considered that the pooled results in men and cohort studies group might be affected by the reported by Wilson et al. (34). This cohort study only included male smokers, and showed that vitamin D intake was positively associated with RCC. Therefore, the finding may not be generalizable to non-smoking populations. The inconsistent subgroup analysis results in our study need to be further validated by future studies.

To our knowledge, the current values to classify vitamin D sufficiency and deficiency are still controversial. Furthermore, the utility of vitamin D status screening versus supplementation strategies still remains surrounded by uncertainties (18). Recently, McCullough et al. pooled 5706 colorectal cancer and 7107 control participants from 17 cohorts, and identify optimal concentrations of circulating 25(OH)D levels (30-40ng/mL) for colorectal cancer risk reduction (53). However, the optimal 25(OH)D levels for RCC reduction remains unclear. In this meta-analysis, we did not examine the dose-response relationship between circulating and dietary intake of vitamin D and risk of RCC due to the small number of included study and lack of dietary intake and circulating vitamin D dose in some studies. We believe that these issues deserve further discussion.

Our study has several strengths. The summary risk estimates of this meta-analysis were based on several large sample studies such as the Nurse's Health Study, Health Professionals Follow-up Study, and Alpha-Tocopherol, Beta-Carotene Cancer Prevention Study. Several studies had a prospective design and long duration of follow-up. No significant heterogeneity was observed between the included studies. Furthermore, most of the included studies were high quality and adjusted for several potential confounding factors such as age, sex, body mass index, smoking status, and energy intake, which increased the accuracy of the pooled results and improved the strength of the evidence. However, the present meta-analysis had several limitations. First, the number of included articles was limited. Only case-control and cohort studies were examined in our meta-analysis because randomized clinical trials in this area are still lacking. Second, we assessed the association between dietary intake of vitamin D and RCC risk, but studies that reported supplemental vitamin D were removed from our analysis because of the potential limitations of the use of vitamin supplements. Finally, to our knowledge, although dietary vitamin D intake accounting for a proportion of circulating vitamin D levels, the circulating vitamin D level is mainly determined by UV radiation from sunlight. However, this part of the exposure cannot be evaluated in this study at present. Therefore, it is needed to be further explored and analyzed in the future observational studies.

## CONCLUSION

Overall, this meta-analysis found that higher dietary vitamin D intake and higher circulating vitamin D level both might be associated with a reduced risk of RCC. In addition, the effects of vitamin D intake were more evident in case-control studies and in women participants. Considering the limitations of the included studies, further high-quality randomized controlled trials are necessary to confirm the results of our study and provide more conclusive evidence.
